# Severe congenital neutropenia caused by *ELANE* gene mutation: A case report and literature review

**DOI:** 10.1097/MD.0000000000031357

**Published:** 2022-11-04

**Authors:** Jing Wang, Haitao Zhang, Yu Wang, Lei Liang, Zeyu Yang

**Affiliations:** a Department of Respiratory, Anhui Provincial Children’s Hospital Affiliated to Anhui Medical University, Hefei, China; b Department of Respiratory, Anhui Provincial Children’s Hospital, Hefei, China.

**Keywords:** *ELANE* gene mutation, *Mycobacterium abscess*, recurrent infection, severe congenital neutropenia, treatment

## Abstract

The Severe congenital neutropenia (SCN) is a rare genetic disease characterized by a deficiency of mature neutrophils in the bone marrow and peripheral blood disorders. After a BALF ACER examination, the patient was found to have a rareMycobacterium abscess infection.

**Patient concerns:**

We report a case of SCN withMycobacterium abscess infection caused by ELANE gene mutation. Conventional antiinfection and granulocyte colony-stimulating factor (G-CSF) did not ameliorate patient’s symptoms. The absolute neutrophil count (ANC) most of the time < 0.50 × 109/L.

**Diagnoses:**

According to Gene sequencing and other tests, the patient was diagnosed with SCN caused by ELANE gene mutation, severe pneumonia, Mycobacteriosis abscess, nutritional iron deficiency anemia, multiple abscesses of the skin, hypergammaglobuloemia, and thrush.

**Interventions:**

Anti-infection agents, abscess incision and drainage, blood transfusion, G-CSF were treated.

**Outcomes:**

The fever subsided, the cough disappeared, the anemia improved, and the ANC improved (0.69 × 109/L). Currently, the patient has been followed up in the outpatient clinic for 20months, during which time fever, bone pain, gingivitis and thrush occasionally appeared. The ANC fluctuated between 0.20 and 1.27 × 109/L, suggesting the need for a timely hematopoietic stem cell transplant (HSCT).

**Lessons:**

*ELANE* gene-related SCN is rare in children, and the possibility of this disease should be considered in children with recurrent severe bacterial infections and a significant reduction in neutrophils in the peripheral blood shortly after birth. In addition to strengthening nursing care and actively preventing and controlling infection, other rare bacterial infections should be considered in clinical practice.

## 1. Introduction

Severe congenital neutropenia (SCN) is a heterogeneous genetic syndrome characterized by a deficiency of mature neutrophils in the bone marrow and peripheral blood. First reported by Swedish pediatrician Kostmann in 1956, it is also known as Kostmann syndrome.^[1]^ At present, the disease has been confirmed to be associated with a variety of gene mutations, mainly occurring in genes such as *ELANE*, *HAX1*, *GFI-1*, *WASP*, *G6PC3*, *CSF3R*, and *JAGN1*, of which 50% to 60% of cases are caused by *ELANE* gene mutations.^[2,3]^ Clinically, the incidence of SCN is extremely low, and children often have repeated serious bacterial infections soon after birth, which will pose a threat to their life and health without proper treatment. In addition, some children are at risk of developing myelodysplastic syndrome (MDS) or acute myelogenous leukemia (AML), which is considered a precancerous condition.^[4–6]^ Therefore, early diagnosis and reasonable treatment of children with SCN are of great significance to improve their prognosis.

Due to the clinical heterogeneity and low incidence of SCN, missed and inaccurate diagnosis are common, resulting poor prognosis. Herein, we report a boy who presents typical clinical manifestation and *Mycobacterium abscess* infection with SCN.

## 2. Case presentation

**Figure 1.**
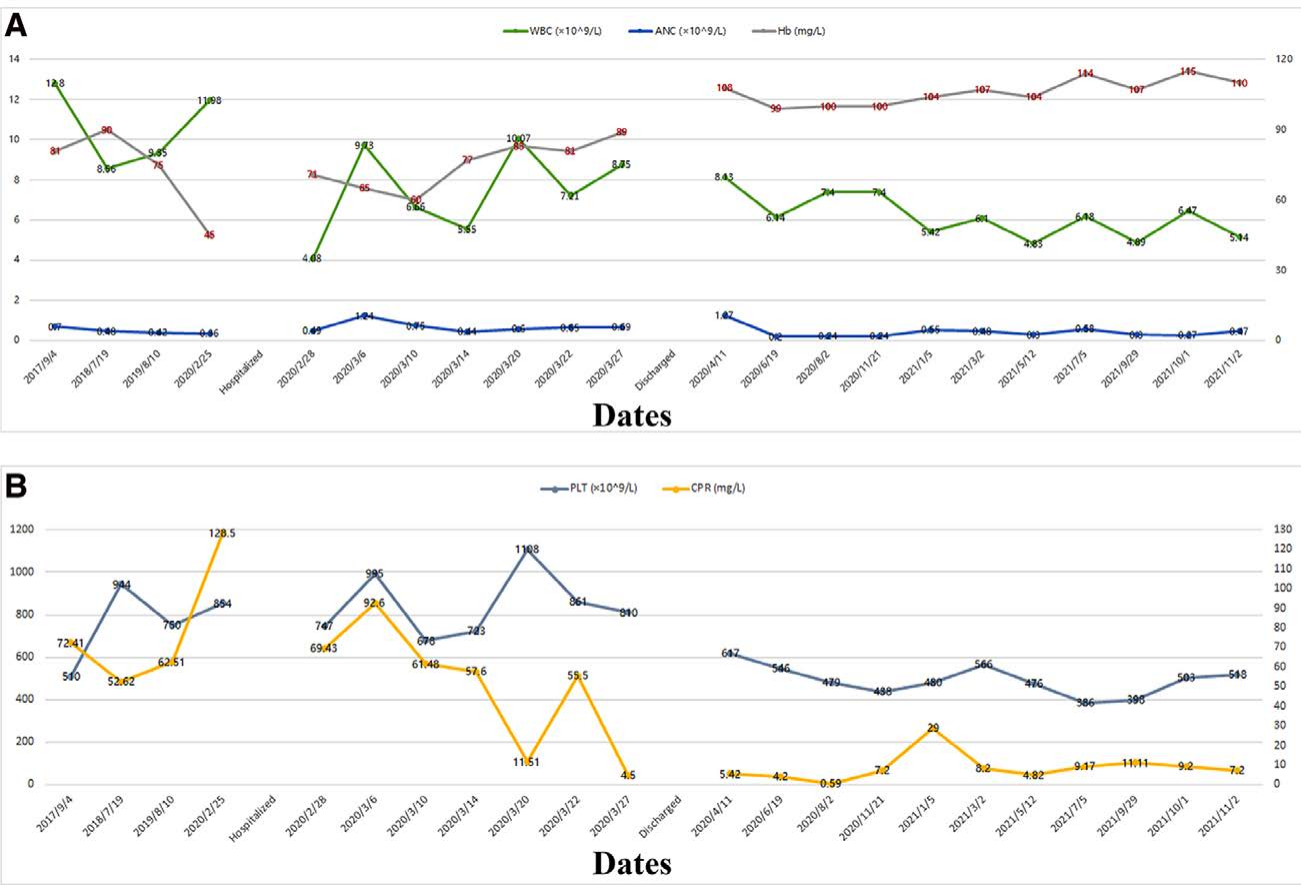
(a) Results of WBC, ANC, and Hb testing at different times; (b) Results of PLT and CRP testing at different times. ANC = absolute neutrophil count, CRP = C-reactive protein, Hb = hemoglobin, WBC = white blood cells.

A 30-month-old male child was admitted to our hospital on February 28, 2020 due to recurrent fever, skin and soft tissue infections for 2 and a half years and cough and mental deficit for 10 days. On the 14th day after his birth, he was diagnosed with “neonatal septicemia, purulent meningitis, skin infection, neutropenia and moderate anemia” in another hospital due to fever and skin and soft tissue infections (occipital and upper arm). He was given symptomatic anti-infection treatment and discharged after his condition improved. After that, he suffered from recurrent fever, skin and soft tissue infections (manifested as abscesses around the anus, occipital, face, opisthenar, upper arm, and armpit), sometimes accompanied by cough, discharge of pus from the external ear canal, and red and swollen gums. He was hospitalized in other hospitals several times, and his symptoms improved after treatment with anti-infection drugs and abscess incision and drainage but soon recurred. Repeated laboratory tests showed that the absolute count of neutrophils (ANCs) was 0.42 to 0.70 × 10^9^/L, hemoglobin (Hb) was 75.00 to 90.00 g/L, C-reactive protein was 52.60 to 72.40 mg/L and platelet was 510.00 to 944.00 × 10^9^/L (Fig. 1a and b). On February 18, 2020, he developed fever, cough and lethargy again and was hospitalized in another hospital. Examination revealed ANC 0.36 × 10^9^/L, Hb 45.00 g/L and C-reactive protein 128.50 mg/L. Chest CT showed extensive inflammatory changes in both lungs and bilateral pleural thickening. After anti-infection treatment (cefoperazone sodium and sulbactam sodium, meropenem, erythromycin), administration of red blood cells, and resection and drainage of the right axillary abscess, his symptoms did not improve, and he was transferred to our hospital.

**Figure 2.**
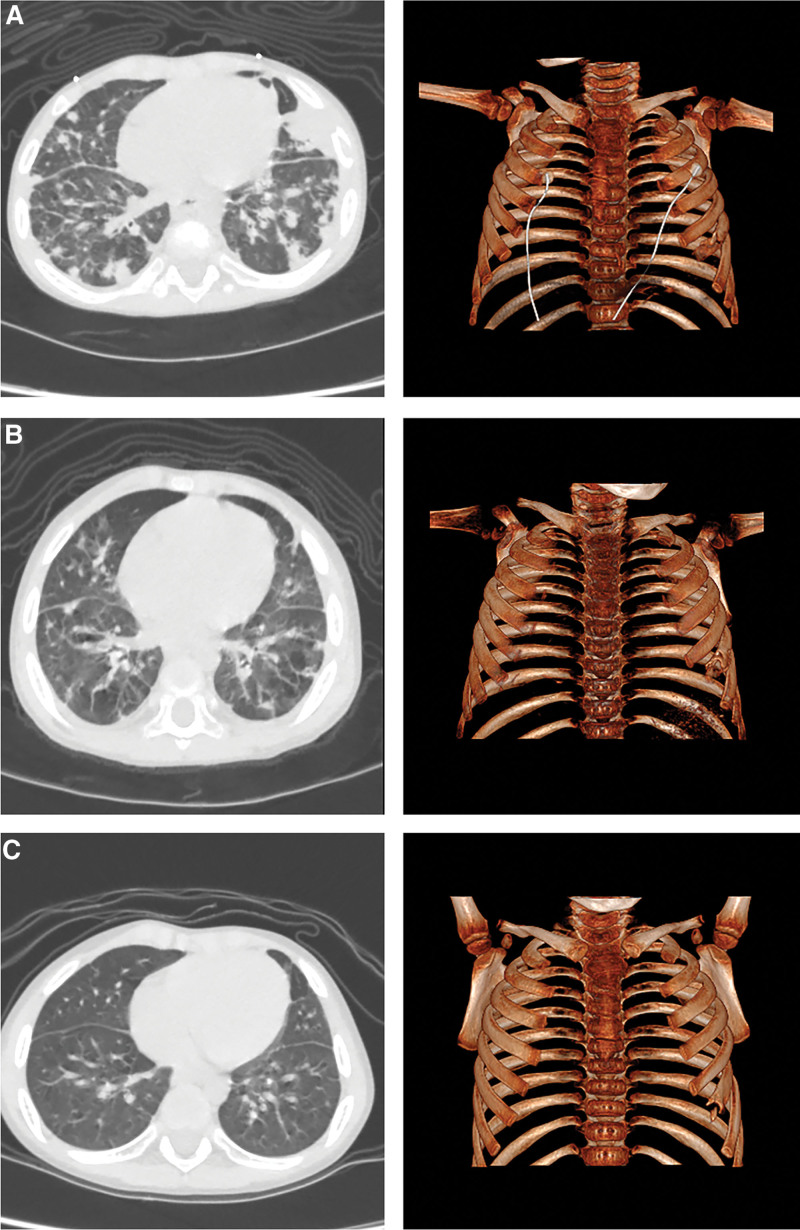
(a) Family pedigree of the patient; (b) Gene sequencing results: The child had an *ELANE* mutation C.125C > T (exon 2), while the parents of the child had normal wild type *ELANE* without heterozygous variation; (c) The *ELANE* missense mutation C.125C > T (exon 2), located on chromosome 19 (19p13.3).

**Figure 3.**
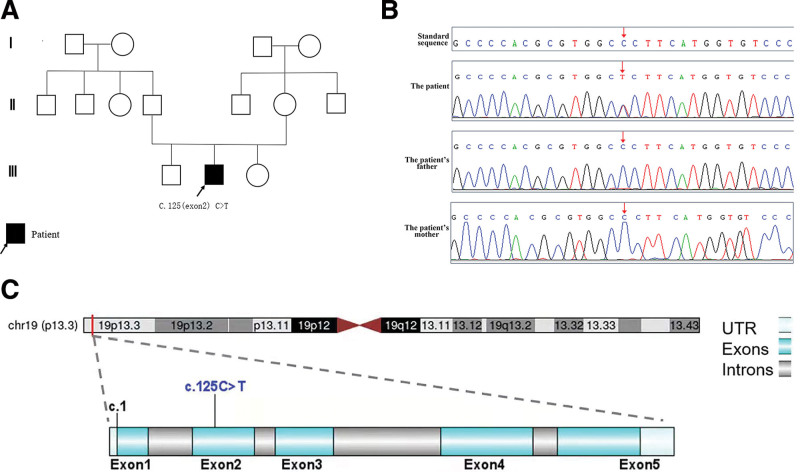
(a) Chest CT showed bilateral diffuse interstitial lesions with multiple small abscesses and nodular high-density shadows, mediastinal and bilateral axillary lymph node enlargement, and bone destruction of the left sixth anterior rib; (b) After hospitalization, the lesions in both lungs were absorbed more than before, but the bone destruction of the left sixth anterior rib was not significantly improved; (c) The absorption of the bilateral lung lesions was significantly better than before, and the bone destruction of the left sixth anterior rib, but remodeled than before.

The child was born at term with a birth weight of 3500 g. His parents, 1 older brother and 1 sister were healthy, and there was no family history of hereditary diseases (Fig. 3a). On admission to our hospital, physical examination exhibited consciousness, spirits somewhat poor; normal growth and nutritional status but anemia; old scars on the right hand middle finger, occipital, right upper arm; abscesses on the left hand back and right ankle; the right axillary abscess incision is unhealed; tachypnea; swollen gums; positive for the 3 concave sign; pharyngeal hyperemia; white curds are seen in the mouth; moist rales in both lungs; and other organs normal. Laboratory examination found ANC <0.50 × 10^9^/L many times (Fig. 1a and b) and normal liver, kidney and heart functions. Total protein 87.7 to 104.9 g/L, albumin 20.7 to 30.1 g/L, and globulin 57.6 to 83.5 g/L. Meanwhile, procalcitonin (PCT, 0.800 ng/mL), (Interleukin-6, 2.830 pg/mL), fungal-specific antigen, serum aspergillus-specific antigen, tuberculin skin test, antineutrophil cytoplasmic antibody, blood culture, sputum culture, and acid fast staining were all negative. Bronchoalveolar lavage fluid culture, cell composition analysis and acid fast staining were negative. Mp-DNA was positive in the bronchoalveolar lavage fluid. MP-Ab-IgM, RSV-Ab-IgM, and influenza B virus Ab-IgM were positive. Immunological evaluation showed IgG 29.77 g/L, IgA 3.05 g/L, and IgM 2.25 g/L. Flow cytometric analysis performed on the bone marrow aspirate showed CD3 + 56.0%, CD3 + CD4 + 32.3%, CD3 + CD8 + 20.3%, and CD3-CD16 + 56 + (NK cells) 6.1%. *Staphylococcus aureus* was cultured from puncture fluid of the skin abscesses. Chest CT revealed diffuse interstitial lesions in both lungs with multiple small abscesses and nodular high-density shadows, mediastinal and bilateral subaxillary lymph node enlargement, and bone destruction of the left sixth anterior costal rib (Fig. 2a). Bronchoscopy revealed inflammation of the trachea and bronchial mucosa. Other examinations had no obvious abnormity.

We made the primary diagnosis as severe pneumonia, neutropenia, nutritional iron deficiency anemia, multiple abscesses of the skin, hypergammaglobulinemia, and thrush. The treatment with cefoperazone sulbactam, linezolid, meropenem, itraconazole, vancomycin, oral care, transfusion of red blood cell suspension and G-CSF (granulocyte colony-stimulating factor) did ameliorate the cough, anemia, thrush and other symptoms. However, the child still had fever, the incision site of the abscess was red and swollen, with pus and blood exudation and subcutaneous induration. Laboratory examination showed ANC <1.00 × 10^9^/L (Fig. 1a). In order to clarify the diagnosis, we performed bone marrow cytology and BALF ACER examination. With the approval of parents, the peripheral blood of the boy and his parents were collected for whole-exon sequencing. Bone marrow cytology showed active myelodysplasia, decreased granulocytic hyperplasia, an increased promyelocytic granulocytic proportion (8.0%), significantly decreased rod-shaped nuclei (1.0%) and lobulated nuclei (2.5%), indicating a bone marrow maturation disorder. The proportion of eosinophils, mature monocytes and plasma cells were increased. Erythroid hyperplasia was active, mainly in the middle and late juvenile erythrocytes, and the central hyperchromatic area of the mature erythrocytes was increased. BALF ACER examination revealed *S aureus* and *M abscess* infection. Whole-exome sequencing yielded a mean of 10 Gb data with >99% coverage of the target region. Compared with reference sequence (hg19), a missense variant, located in chr 19: p13.3, was detected in exon 2 of *ELANE* [c.125 C > T (p.P42L); NM_001972;] in the boy (Fig. 3b and c). In terms of Variant annotation and pathogenicity prediction, we used an on-line system independently developed by Chigene. Chigene (www.chigene.org) was used to annotate the database-based minor allele frequencies (MAFs) and ACMG (American College of Medical Genetics) practice guideline-based pathogenicity for every gene variant. The system also provided serial software packages for conservative analysis and protein product structure prediction. The databases for the annotation of MAFs include 1000 genomes, dbSNP, ESP, ExAC, and Chigene in-house MAFs database; Provean, Sift, Polypen2_hdiv, Polypen2_hvar, MutationTaster, M-Cap, and Revel software packages were used to predict protein product structure variation. The prioritized pathogenicity annotation of ACMG guidelines and the OMIM databases were used as conferences for pathogenicity of every variant. No variant was detected in the patient’s parents and their genotype was normal (Fig. 3b). Sanger sequencing was used to verify the de novo variant in the proband and her parents. According to the 2015 ACMG guidelines, the variant meets the criteria to be identified as a pathogenic variant: PS1 + PS2 + PM2 + PP3. According to the above examination results, the patient was finally diagnosed with SCN and *M abscess*.

Regarding his multiple abscesses of the skin and *M abscess*, abscess incision drainage was performed, along with amikacin and cefoxitin. Considering that the child had rib bone damage, we did not increase the dose of G-CSF, but continued to give 5 μg·kg^−1^·d^−1^. Subsequently, the patient’s body temperature was normal on the 25th day after admission. Chest CT reexamination showed that the absorption of the lung inflammation had improved (Fig. 2b), and the laboratory examination showed ANC 0.69 × 10^9^/L and the incision on his abscess has healed, so the patient was discharged after being hospitalized for 29 days. After discharge, oral linezolid, azithromycin and itraconazole for infection prevention, dipyridamole for platelet aggregation prevention and subcutaneous injection of G-CSF were recommended (G-CSF was 10–15μg/kg per day, once a day). The patient was followed up in the clinic for 20 months, during which time fever, bone pain, gingivitis and thrush occasionally appeared. The ANC fluctuated in the range of 0.20 × 10^9^/L to 1.28 × 10^9^/L, most of the time <0.50 × 10^9^/L, Hb: 99.00 g/L to 115.00 g/L, PLT: 398.00 × 10^9^/L to 617.00 × 10^9^/L (Fig. 1a and b). Twelve months after discharge, chest CT reexamination showed that the absorption of the double lung lesions was significantly better than before, but there was still bone destruction in the ribs (Fig. 2c).

## 3. Discussion and conclusions

We searched Chinese and English databases (PubMed, CNKI, WanFang database) for “Severe Congenital Neutropenia” from its establishment to November 20, 2021. A total of 626 articles were retrieved, including 99 articles on severe congenital neutropenia caused by *ELANE* gene mutations. Eighty-five papers were excluded because of duplicates and cases with an incomplete medical history. The clinical data of 17 children were summarized and analyzed, excluding repeated reports and incomplete histories (Table 1). There were 8 boys and 9 girls. Their ancestry was Chinese (n = 3), Indian (n = 3), Korean (n = 5), American (n = 4), Greek (n = 1), or Vietnamese (n = 1). The 17 patients had 15 different mutation sites. Patient #14 and patient #15 were all mutated at c.302T > G, p.V101G. Both patient #10 and patient #11 had mutations at c.597 + 1 G > A. Patient #14 was related to patient #15, and they both had the same genetic mutation. Patient #10 was related to patient #11, and they both had the same genetic mutation. The main clinical manifestations of the patients were recurrent infections (17/17, 100%). Fifteen patients were treated with G-CSF and antibiotics, eleven of whom responded well to treatment, with increased WBC (white blood cells) and decreased numbers of infections (11/15, 73%). After patients #14 and #15 received HSCT (hematopoietic stem cell transplant) treatment, their WBC and ANC remained at normal levels without recurrence of infection.

**Table 1:** Clinical features of severe congenital neutropenia caused by ELANE gene mutation.

#	**A**ge at diagnosis	Country	Sex	Mutation	Clinical signs and symptoms	Treatment	Response to G-CSF	References
1	2.6 yr	China	Male	c.125C>T	Skin infections, pneumonia, otitis media	G-CSF, antibiotics	(−)	This report
2	4 yr	China	Female	c.661G>T	Periodontitis, pneumonia	Antibiotics	Did not use	^[7]^
3	2.9 yr	China	Male	c.290A>C	Recurrent upper respiratory infection, mycotic stomatitis, a perianal abscess, pneumonia	Antibiotics	Did not use	^[8]^
4	3 yr	India	Female	c.201T>A	Skin infection, upper respiratory tract infection	G-CSF, antibiotics	(−)	^[9]^
5	1.8 yr	India	Male	c.457G>C p.A153P	Dyspnoea, otitis media, ulcers in right parotid, perianal, regionspneumonia	G-CSF, antibiotics	(+)	^[10]^
6	3 mo	India	Male	c.215T>A p.V72G	Poor abdominal scar healing, pneumonia, omphalitis, ischiorectal abscess.	G-CSF, antibiotics	(+)	^[11]^
7	1.2 mo	South Korea	Male	c.658delC p.A220G	Otitis media	G-CSF, antibiotics	(+)	^[12]^
8	7 yr	South Korea	Male	c.170C>A p.A57A	Cervical adenopathy	G-CSF, antibiotics	(+)	^[13]^
9	9 mo	South Korea	Female	c.607G>C p.G203A	Cervical lymphadenitis	G-CSF, antibiotics	(+)	^[14]^
10	1.5 yr	South Korea	Female	c.597 + 1G>A	Recurrent fever, cervical lymphadenitis	G-CSF, antibiotics	(−)	^[15]^
11	3.1 yr	South Korea	Female	c.597 + 1G>A	Recurrent oral ulcers	G-CSF, antibiotics	(−)	^[15]^
12	2 yr	United States	Female	p.G214 V	Otitis media, cellulitis	G-CSF, antibiotics	(+)	^[16]^
13	10 mo	United States	Male	c.356T>A p.V119E	Omphalitis, recurrent bacterial infection	G-CSF, antibiotics	(+)	^[17]^
14	4 yr	United States	Female	c.302T>G p.V101G	Pyoderma, stomatitis, pneumonia, cervical lymphadenitis, chronic gingivitis	G-CSF, antibiotics, HSCT	(+)	^[18]^
15	10 mo	United States	Female	c.302T>G p.V101G	Stomatitis, cervical suppurative lymphadenitis, pneumonia	G-CSF, antibiotics, HSCT	(+)	^[18]^
16	6 yr	Vietnam	Male	c.242G>C	Cutaneous abscess behind right ear, pustulosis on skin, foot fungus, mouth ulcer	G-CSF, antibiotics	(+)	^[19]^
17	5 yr	Greek	Female	c.157C>G p.H53A	Pneumonia, repeated bacterial infections	G-CSF, antibiotics	(+)	^[20]^

G-CSF = granulocyte colony-stimulating factor, HSCT = hematopoietic cell transplantation.

SCN is a rare disorder of hematogenesis with a very low incidence of approximately 3 to 8.5/1 million.^[3,21]^ The mode of inheritance is a single gene mutation, which can manifest as autosomal recessive inheritance, autosomal dominant inheritance, X chromosome linkage inheritance and sporadic disease without any obvious genetic background.^[3]^ Autosomal dominant inheritance, usually associated with defects in the *ELANE* gene. The *ELANE* gene, located on chromosome 19 (19p13.3), consists of 5 exons and 6 introns encoding a neutrophil elastase (NE) of 218 amino acids.^[22]^ This enzyme is a myeloid cell-specific serine protease that is produced during promyelocytic differentiation of neutrophils and it exists in the primary granules of mature neutrophils.^[23]^ It can hydrolyze a variety of extracellular matrix and bacterial components, such as elastin, and it plays an important role in innate immune defence.^[2]^ Different hypotheses have been proposed to explain the pathogenicity of *ELANE* mutations. The most widely accepted notion is that mutant NE triggers apoptosis of developing neutrophils by initiating the unfolded protein response.^[24,25]^ To date, >120 mutation sites have been reported, including missense, transcoding, nonsense, intron splicing, insertion and deletion.^[24,25]^ Screening results of immunodeficiency disease-related genes in this case showed that the child had an *ELANE* missense 2c.125C > T (Fig. 3b and c). The child was heterozygous de novo mutation, which was consistent with the pathogenesis of autosomal dominant genetic diseases.

SCN is clinically characterized by persistent severe neutropenia (<0.50 × 10^9^/L), recurrent bacterial infection, and maturation arrest in the bone marrow.^[12]^ Bacterial infections involving *S aureus* and epidermidis, streptococci, enterococci, pneumococci, *Pseudomonas aeruginosa* and Gram-negative bacilli.^[26]^ The infection was the dominant clinical symptom (17/17, 100%), as proposed in Table 1. In this case, children suffered from neonatal pneumonia, perianal soft tissue infections, sepsis, purulent meningitis and so on shortly after birth, and in the infant stage, he often showed repeated fevers, skin abscesses, gingivitis, oral ulcers, otitis media and so on. As physicians of respiratory, we paid more attention to anti-infection treatment of the patient. Reports indicate that the key to the treatment of SCN caused by *ELANE* gene mutation is infection prevention.^[3,4]^ Therefore, after admission, we gave a combination of antibiotics, but the child still had recurrent fever, we performed BALF ACER examination. Through BALF ACER examination, we found that the patient was accompanied by multiple bacterial infections, among which *M abscess* was very rare and had multiple drug resistance.^[27]^ This is the first CASE of SCN with *M abscess* infection in China. *M abscess* is a kind of nontuberculous mycobacterium. It can cause a wide spectrum of human disease, most commonly pulmonary disease, but also soft tissue disease, bone disease, and disseminated disease in immunocompromised hosts.^[28]^ The patient’s Chest CT showed diffuse interstitial lesions in both lungs with nodular high-density shadows, and clinical symptoms of cough, emaciation, malaise and fever, which are typical nontuberculous mycobacterium lung diseases. However, acid-fast staining was negative, so this diagnosis was not considered in the early stage, resulting in unsatisfactory anti-infection treatment effect. After adding amikacin, cefoxitin, the lung infection and fever were significantly improved. In addition to the pulmonary manifestations, the incision of the skin abscess was red and swollen, accompanied by pus and blood secretions and subcutaneous hard nodes. It was considered as soft tissue disease caused by *M abscess*. So, we suggested pathological biopsy for further diagnosis, but the parents refused. Abscess incision drainage was performed, along with amikacin and cefoxitin, the incision on his abscess healed gradually.

Since the first clinical application of G-CSF in the 1980s, the treatment of this disease has made a breakthrough, and the prognosis and quality of life of patients have been greatly improved.^[4,5]^ In addition to anti-infective therapy, G-CSF is the first-line drug in the treatment of SCN, and it has the effect of promoting neutrophil generation and reducing and controlling infections.^[6]^ Studies have shown that >90% of patients are responsive to G-CSF treatment.^[3]^ Fifteen patients were treated with G-CSF, eleven of whom responded well to treatment (Table 1), with increased WBC and decreased numbers of infections (11/15, 73%). The required dose of G-CSF varies widely. Generally, the initial dose is 5 μg·kg^−1^·d^−1^, subcutaneously injected daily, increased by 5 μ/kg every 3 to 5 days until effective, and ANC >1.00 × 10^9^/L is maintained.^[14,15]^ Most SCN patients responded to a dose between 3 and 10 μg·kg^−1^·d^−1^. When the G-CSF dose exceeds 50 μg·kg^−1^·d^−1^ and is ANC was still <0.50 × 10^9^/L, patients are considered to have no response to G-CSF.^[3–6]^ To date, no effective treatment has been developed for patients who are nonresponsive or resistant to G-CSF. HSCT is currently the only treatment option for patients who do not respond to G-CSF and those who have developed MDS/AML.^[29,30]^ During the follow-up period of 12 months after discharge, the G-CSF (10 μg·kg^−1^·d^−1^) was still used for treatment in this case, but ANC was <0.50 × 10^9^/L most of the time. So, it was considered that the child had a poor response to G-CSF. The French Registry of Severe Chronic Neutropenia, Severe Chronic Neutropenia International Registry, and a study in the Swedish population reported that the cumulative incidence of MDS/AML in SCN patients was 22.0%, 8.1%, and 31.0%, respectively, 15 years after G-CSF initiation.^[4–6]^ In addition to increasing the risk of progression to MDS/AML, G-CSF also includes complications such as bone pain, splenomegaly, thrombocytopenia and osteoporosis, according to the Severe Chronic Neutropenia International Registry.^[9]^ Therefore, considering the side effects caused by high dose and long-term use of G-CSF, we consulted the parents and increased the dose of G-CSF (15 μg·kg^−1^·d^−1^) with the consent of the parents. However, during the 8-month follow-up period, ANC did not increase and remained <0.50 × 10^9^/L. Meanwhile, the patient developed bone pain, and chest CT reexamination showed that there was still bone destruction in the ribs (Fig. 2c). Studies have shown that when G-CSF dose reaches 15 μg·kg^−1^·d^−1^ and ANC is <0.50 × 10^9^/L, HSCT is considered to reduce the risk of developing MDS/AML. Therefore, HSCT was suggested in child rather than increasing the dose. The parents agreed with the proposal.

In conclusion, *ELANE* gene-related SCN is rare in children, and the possibility of this disease should be considered in children with recurrent severe bacterial infections and a significant reduction in neutrophils in the peripheral blood shortly after birth. The diagnosis mainly depends on genetic testing. In addition to strengthening nursing care and actively preventing and controlling infection, other rare bacterial infections should be considered in clinical practice. G-CSF is an effective treatment. However, it is important to pay attention to the side effects caused by long-term use of G-CSF. HSCT can be considered in patients with no response to G-CSF and among patients with MDS/AML transformation.

## Author contributions

ZY designed the study. HZ and JW performed the literature search and review, wrote the manuscript, and critically revised it. ZY critically reviewed the manuscript, YW and LL contributed to modify the manuscript. All authors contributed to the article and approved the submitted version.

Conceptualization: Zeyu Yang.

Data curation: Yu Wang, Lei Liang.

Supervision: Lei Liang, Zeyu Yang.

Writing – original draft: Jing Wang.

Writing – review & editing: Haitao Zhang, Zeyu Yang.
